# Implementing the first regional hospice palliative care program in Ontario: the Champlain region as a case study

**DOI:** 10.1186/s12904-016-0131-6

**Published:** 2016-07-26

**Authors:** José Pereira, Jocelyne Contant, Gwen Barton, Christopher Klinger

**Affiliations:** 1 Champlain Hospice Palliative Care Program, 69 Primrose Avenue, Annex C, Saint-Vincent Hospital, Ottawa, Ontario K1R 6M1, Canada; 2 Division of Palliative Care, Department of Medicine, University of Ottawa, 501 Smyth Road, Ottawa, Ontario K1H 8L6, Canada; 3 Department of Palliative Medicine, Bruyère Continuing Care, 43 Bruyère Street, Room 270-J, Ottawa, Ontario K1N 5C8, Canada

**Keywords:** Regional, Palliative care, Change management, System

## Abstract

**Background:**

Regionalization promotes planning and coordination of services across settings and providers to meet population needs. Despite the potential advantages of regionalization, no regional hospice palliative care program existed in Ontario, Canada, as of 2010. This paper describes the process and early results of the development of the first regional hospice palliative care program in Ontario. The various activities and processes undertaken and the formal agreements, policies and documents are described.

**Methods:**

A participative approach, started in April 2009, was used. It brought together over 26 health service providers, including residential hospices, a palliative care unit, community and hospital specialist consultation teams, hospitals, community health and social service agencies (including nursing), individual health professionals, volunteers, patients and families. An extensive stakeholder and community vetting process was undertaken that included work groups (to explore key areas such as home care, the hospital sector, hospice and palliative care unit beds, provision of care in rural settings, e-health and education), a steering committee and input from over 320 individuals via e-mail and town-halls. A Transitional Leadership Group was elected to steer the implementation of the Regional Program over the summer of 2010. This group established the by-laws and details regarding the governance structure of the Regional Program, including its role, responsibilities, reporting structures and initial performance indicators that the Local Health Integration Network (LHIN) approved.

**Results:**

The Regional Program was formally established in November 2010 with a competency-based Board of 14 elected members to oversee the program. Early work involved establishing standards and performance indicators for the different sectors and settings in the region, and identifying key clinical needs such as the establishment of more residential hospice capacity in Ottawa and a rural framework to ensure access for citizens in rural and remote regions. Challenges encountered are explored as are the process enablers and facilitators. The paper views the development and implementation process from the perspectives of several frameworks and models related to change management.

**Conclusions:**

Following on several initial achievements, the long term success of the Regional Program will depend on consolidating the early gains and demonstrating changes based on key measurable outcomes.

## Background

A regional approach to health systems design has yielded significant successes in the provision of palliative care services, including hospice and end-of-life care. Jurisdictions such as Edmonton, Alberta in Canada [1, 2] and Catalonia and Extremadura in Spain adopted such an approach in the 1990’s [3, 4]. Results have included improved access to palliative care services in the community and hospitals, more cancer patients dying in their homes or hospices instead of acute care hospitals, increased capacity in the primary care sector to provide palliative care and reduced use of emergency departments. Improved access and quality have been accompanied by significant cost savings for the respective health care systems in these jurisdictions.

Regionalization promotes a broader approach to health systems design; rather than focusing on individual providers and institutions, it improves planning and coordination of services across settings and providers to meet population needs [5]. At its core is the recognition that health care systems are generally complex and adaptive, requiring systems-based thinking to develop and manage them. Complex systems have many components - including patients, families of patients, health professionals, service providers, policy makers, administrators and funders - and these are continuously interacting and adapting to changes in other components and in the environment [6]. The distinctive features of these systems include self-organization, constant changes, non-linearity, time lags between inputs and outcomes, a past history and unintended consequences of previous policy interventions [7]. Complex systems continuously adjust in dynamic and sometimes unpredictable ways in response to changes in the environment in which they operate.

In November 2010, the first regional palliative care program in the Canadian province of Ontario was established in the Champlain Region. This paper describes the context for developing the Regional Program and the roadmap taken. The paper reflects on the process, using existing frameworks of change-management and systems-implementation, to explore what was done well, identify what could have been done differently and share lessons learned.

## Methods

The Canadian health system is based on universal access for all residents, but is de-centralized in that provincial governments are constitutionally empowered to deliver health services within their respective jurisdictions [8]. Therefore, four levels of health service planning and delivery exist: national, provincial, regional and local. This has resulted in differences in health services between provinces and even between regions within a province.

In 2007, the Ontario Ministry of Health and Long-Term Care (MoHLTC) created organizations called Local Health Integration Networks (LHINs) to oversee health services in each of 14 geographical regions across the province [9]. They are responsible for, amongst others, funding and monitoring hospitals, homecare (including Community Care Access Centres or CCACs) and community support services. The LHIN structure is intended to be a mechanism for overcoming existing health care “silos” and improving integration and coordination of services regionally.

Unlike regional health authorities in other Canadian provinces, LHINs do not directly provide care services. Instead, they fund organizations (known as health service providers or HSPs), to provide the services [10]. Service providers maintain their own governance, but enter into contracts called “Service Accountability Agreements” with the LHINs. These agreements set budgets and expectations regarding the scope, nature and volume of services to be provided (managed competition process). The MoHLTC retains control of strategic policy-making direction and standards setting for Ontario’s health system.

The Champlain LHIN region is situated in south-eastern Ontario. It covers 18,000 km^2^, is about 260 km in length and has 1.2 million inhabitants; 70 % of the population lives in the Ottawa area, the largest city in the region with a population of approximately 980,000. Twenty percent of the region is Francophone and there are 32,000 Aboriginal peoples living off reserves. There are over 200 service providers in the region. With the exception of the Ottawa area, most of the region is rural and consists of towns, villages, farms and wooded areas [11].

Palliative care services and programs, including hospice services, are provided by numerous organizations. Some, such as CCACs (the service that coordinates home care) and hospitals, are formally recognized as HSPs. Others, such as nursing agencies (that provide the hands-on home care) and hospices are generally not. In the absence of a single regional health service provider, service planning and provision are done by the individual service providers. This often results in planning and funding requests to the LHIN that are service provider-centric, with limited collaboration and planning between different service providers for a common regional goal.

Over the years, the MoHLTC has implemented several palliative care initiatives. These have included small, nurse-only support teams in the community to support primary care professionals and capital funding (but limited operational funding) to build small free-standing residential hospices. In 2005, under the auspice of the Ontario End-of-Life Care Strategy, the Ministry established End-of-Life (EOL) Networks in each region to promote collaboration amongst hospice palliative care service providers. Funding was limited to a coordinator in each region, with no other operational budgets. In Champlain, the region was further subdivided into three zones, referred to as the “Local Networks”. Each was linked informally to the Champlain EOL Network. Notwithstanding these efforts, there has been little over the years in terms of a coordinated strategy in the province, resulting in piece-meal development of palliative care services across the regions.

In 2009, the Champlain EOL Network decided to develop a regional program to improve integration and coordination of hospice palliative care services. The few available system performance indicators for palliative care showed, for example, that 42 % of cancer patients were visiting emergency departments in the last two weeks of life and 52 % of cancer patients were dying in acute care hospitals in Champlain [12]. Long wait times were noted for patients in acute care hospitals waiting for beds in the hospices and the regional palliative care unit - up to a mean of 11.1 days in the region’s largest hospital (unpublished data, The Ottawa Hospital Supportive and Palliative Care Program Internal Database).

**Fig. 1:**
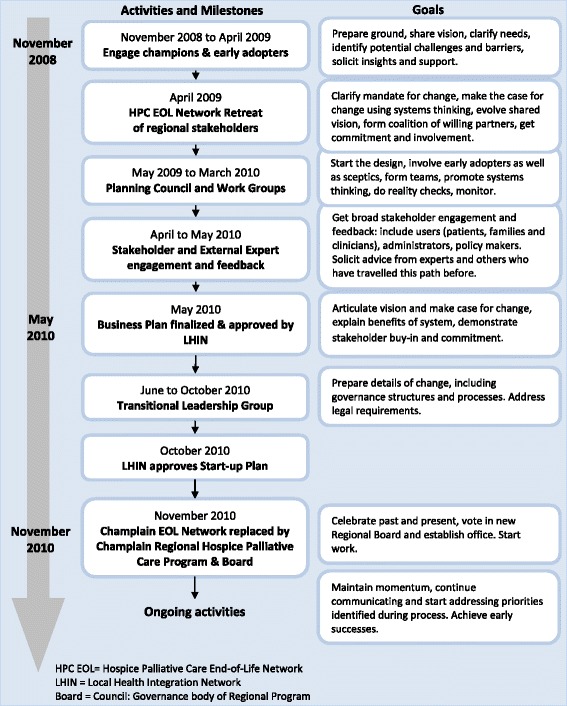
Process for the Planning and Implementation of the Champlain Hospice Palliative Care Program

Led by two of the authors (JP, JC), the EOL Network started the preliminary work of moving towards regionalization in early 2009. Figure 1 summarizes the work and timelines, from the beginning of the process to the inauguration of the new Regional Program.

The first step was to receive an official mandate and support from the LHIN. Key individuals, felt to be potential champions, early adopters and influential decision-makers, were identified. These individuals came from different disciplines and sectors and included the LHIN’s Chief Executive Officer. Formal and informal meetings were held with them to solicit their support and receive advice on the vision. Having formed this support base, the EOL Network convened a day-long retreat in April 2009 of all the stakeholders in the region.

The goal of the April 2009 retreat was to gain wider input and support for regionalization, to begin the process of developing a common vision and to identify priority areas and the next steps. Over 120 individuals and representatives of the service providers and stakeholders directly or indirectly involved in providing hospice palliative care in the region were invited, 70 of which attended. Different settings were represented as were various domains and disciplines (including clinicians, administrators, policy makers, patients and family members). A survey of all the services and programs in the region, based on an Appreciative Inquiry approach [13], was conducted and the results shared at the retreat. A literature review of regionalization and results from jurisdictions that had implemented regional programs were presented, including key components and enablers of such an approach. These included:

- A common vision;
- Accountability processes;
- Performance measurement; and
- A single governance structure. The latter had been identified during the retreat’s preparatory process as a potential barrier to regionalization (particularly given the relative independence of each service provider).

Participants identified several priority areas: a) hospice and palliative care beds for the region; b) consultation services in the community and the hospitals; c) primary care involvement in providing palliative care; d) patient flow and transitions between settings; e) information for the public and health care professionals; f) e-Health; and g) hospice palliative care in rural and remote areas.

The need for a process to help advance the concept of a regional program was identified. The goal was to produce a business plan that would detail what the Regional Program would do, how it would do it, how it would function and what funding support it would require. A report of the retreat was submitted to the LHIN along with a request for $30,000 of funding, largely for administrative and logistical support, to move the above process forward. In May 2009 the LHIN approved the request.

An inter-disciplinary Planning Council, made up of ten palliative care (including hospice) leaders in the region, was established in May 2009. This was co-chaired by two of the authors (JP, JC). The Council was tasked with developing a final business plan for a formal regional program.

Six Working Groups were formed, each focusing on one of the priority areas identified at the retreat. Each group was co-chaired by two Planning Council members and had six to ten members; members were invited based on their expertise and knowledge of the respective areas. A modified Appreciative Inquiry process [13] was used. Groups were asked to discover their successes and existing strengths, to create a vision and to make recommendations on improving each of their areas. Almost 100 individuals participated in the activities of the Planning Council and Work Groups. The Planning Council monitored the work of the Groups and, using a “regional lens”, identified areas of overlap between the groups as well as links and horizontal themes across all groups. Supporting data were collected, including the number of referrals to the various services in the region, lengths of stay in palliative care-related services, wait times for hospices and the palliative care unit and the costs of caring for patients in acute care hospitals waiting for admissions to these hospices and the unit. A full time Coordinator (GB) provided critical support throughout the whole process, from these initial steps to the launch of the Regional Program.

The Planning Council produced a draft Business Plan in March 2010. The Plan proposed, among other things, guiding principles for the process and a new regional program, a governance structure and foundational recommendations. The draft Business Plan was then distributed for broader community input.

Numerous facilitated sessions, using town-hall meeting formats and focus groups, were conducted from April to June 2010 to present the draft Business Plan and to solicit input. In addition to obtaining further feedback, the goal was also to gain broader support for the proposed Program. Over 320 individuals throughout the communities of the Champlain region participated as well as several agencies and societies, including the Champlain chapter of the Canadian Cancer Society and the Amyotrophic Lateral Sclerosis Society. Participants included 55 patients, family members and volunteers, 120 individuals from Community of Care Advisory Forums (groups previously established by the LHIN throughout the region to provide input on health care planning) and 60 clinicians and members from the Francophone communities in the region. The meetings were facilitated by members of the Planning Council. In addition, an online process was established for further feedback (39 individual responses were received) and the draft plan was submitted to four external experts, in jurisdictions outside Ontario that had regional programs, for their insights.

This feedback guided further modifications to the Business Plan. Table 1 lists the key elements in the Business Plan. The Plan contained three foundational recommendations (including the dissolution of the Champlain EOL Network to be replaced by a new Regional Program) and a draft Accountability Agreement between the LHIN and the Regional Hospice Palliative Care (HPC) Program [now Champlain Hospice Palliative Care Program (CHPCP)] (referred to as the LHIN-Regional HPC Program Memorandum of Agreement or MOA). The MOA outlines the reporting relationships with the LHIN and the role and expectations of the Regional HPC Program and of the LHIN. The former is a key document as, given the absence of a single regional health authority and single service provider, it provides the Regional Program with the mandate and legitimacy to exercise its regional function. Key amongst these is that the LHIN Board is to vet all palliative care (including hospice) proposals through the Regional Program and that the Regional Program is to set the priorities for the LHIN’s palliative and hospice related work plans. Table 2 lists the guiding principles for the Program and the foundational recommendations.

**Table 1:** Key elements in the business plan for the Champlain Hospice Palliative Care Program

Background Information
Definitions of palliative care and hospice care
Real life case examples describing patient journey experiences across diagnoses, illness trajectories, needs and settings
Evidence from other jurisdictions of the impact of regional programs and support of regionalization
Key components of successful regional programs internationally
Listing and description of current hospice palliative care services in Champlain
The Planning Process that led to the development of the Business Plan
Regional Supporting Data
Expected population growth and causes of death
Percentage of cancer patients dying in acute care hospitals in the region^a^
Percentage of cancer patients visiting emergency departments in last two weeks of life^a^
Total number of days patients spent waiting in Ottawa hospitals for admission to the Palliative Care Unit or hospice
Mean and median number of days waiting for admission to a PCU or hospice
Total number of new referrals and admissions to the Palliative Care Unit, hospices, home care services, hospital consultation teams
Total number of PCU beds in region
Total number of hospice beds in region
Total number of Long Term Care (LTC) facilities and beds
Provincial Supporting Data
Costs of EOL care in Ontario
Study of emergency department use by cancer patients in last 2 weeks of life
Regulatory, legislative and policy barriers
Recommendations
Foundational, Priority and Supporting recommendations
Guiding principles for Program Development
Program Implementation Plan
Governance Structure
Priority tasks
Evaluation framework with outcomes
Terms of Reference^b^
Timelines
Budget And Item Justifications
Proposed budget items were:
Executive members: Executive Director (full time), Medical Lead (part-time; 0.2 FTE); Decision Support/Informatics Coordinator (full time); Quality Improvement Coordinator (part-time); Local Network Support Personnel (1.5 FTE); Administrative Assistant (full time), operating expenses (translation, meeting & travel expenses, office rental, office supplies, etc.)
Appendices
Working Group recommendations

^a^Source of information: Cancer Care Ontario [8]

^b^Terms of reference for the Program’s Board, Members, Committees, executive officers (Executive Director, Medical Director, Project Manager, Data Manager and Administrative Assistant) were added later

**Table 2:** Guiding principles and foundational recommendations in the business plan for the Champlain Hospice Palliative Care Program

Guiding Principles and Elements
• A common region-wide vision and mission
• A single common governance body that still allows for independence of the various service providers
• An adequately resourced program and system
• Evidence-guided care and diffusion of best practices through education and knowledge transfer
• An accountability system of reporting and system-wide (macro) and institutional (micro) performance indicators
• The establishment of standards for the region
• Improve the capacity of primary care to provide primary-level palliative care (palliative care approach), with adequate resources to provide support to primary care clinicians
• Ongoing role for the local End-of-Life Networks to enhance the role of the Regional Program
Foundational Recommendations^a^
• Establish a Regional Hospice Palliative Care (HPC) Program
• Establish a Program Council of Directors (later renamed “Board of Directors”) to oversee the Program, supported by an executive office
• Establish formal agreements between the Regional HPC Program and the LHIN, and between the LHIN and service providers to: o Support the objectives of the program o Report on key performance indicators

^a^There were also several supporting recommendations covering various priority areas

The Regional Plan was approved by the LHIN’s Board of Directors in May 2010. The Champlain EOL Network was asked by the LHIN to establish a Transitional Leadership Group to oversee the transition between the dissolution of the EOL Network and the inauguration of the new Regional Board.

**Fig. 2:**
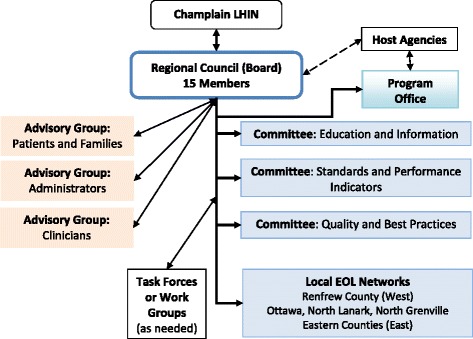
Governance Structure of Champlain Regional Program (2010–2014)
- An annual budget ($300,000);
- By-laws and governance protocols for the inaugural governing body (Board);
- A Work Plan, based on the most pressing priorities, for Year One of the Program’s operations;
- The competency grid of skills required for the Program’s Board. The competencies include those required of each individual member and the collective competencies needed of the Board. Individual competencies include the ability to think “systems” (putting what is best for patients and the system ahead of what is best for an individual service provider). Collectively, as a Board, the domains of competencies include expertise in Systems Development, Governance and Leadership, Project Management and Quality and Performance;
- The election process of the Regional Program’s first Board members.

In June 2010, a Transitional Leadership Group was formed of individuals with the specific skill sets required to oversee the transition, including expertise on governance. Some group members had been members of the now-dissolved Planning Council. The group also received input from the Champlain Regional Stroke Network and Regional Geriatric Program of Eastern Ontario, so that the Transitional Group could benefit from their insights. The Transitional Group developed the following:

- The Terms of Reference for the Regional Program and its committees and members;
- A protocol outlining the accountabilities of a hosting agency or agencies (provincial legislation requires that funds can only be transferred by the LHIN to formally identified HSPs and the Regional Program would not be an HSP);
- The governance structure of the Regional Program (See Fig. 2 which shows the governance structure as of mid-2013);

An open, region-wide process of nominations was initiated; 30 applications were received and 11 members were elected to the new Board.

The Transitional Leadership Group’s work was approved in November 2010 by the Champlain LHIN. The EOL Network was dissolved and the new Champlain Hospice Palliative Care Program (CHPCP, simply referred to as the “Regional Program” in this paper) and a governing Board was inaugurated.

The inaugural structure consisted of the Program Board of Directors (which was initially referred to as the Regional Program’s Council of Directors, but changed to “Board” after incorporation of the Regional Program in 2013) and an office with an Executive Director (ED, full time), Medical Lead (half day a week) and Administrative Assistant (full time). The Board, which consists of 15 members (4 are ex officio), reports to the Champlain LHIN. The Executive Director, Medical Lead and representatives of the two host agencies sit as ex officio members (non-voting). The Medical Lead reports to the ED. The Board is competency based; members do not represent their respective organizations, but are elected to apply patient-centred, systems-based thinking.

Two host agencies were identified: a) a hospital (Bruyère Continuing Care) renting office space and providing back-office support functions; and b) the CCAC providing meeting facilities, telecommunication and outreach to the community.

The inaugural Board had three standing committees (referred to as Specialty Committees): a) Education and Knowledge Translation; b) Performance Management and Access to Services; and c) Standards and Best Practices. The structure maintained the three Local EOL Networks as these provide the means for the Regional Program to implement programs locally and receive feedback and input from local institutions and individuals. There are some funds to support activities in the three Local EOL Networks. Task Forces or Work Groups are constituted as required and are term-limited. The Board has three advisory bodies to provide input to the Program on various issues: a) a patients and families group; b) a health care managers group (who provide perspectives on the different settings, from home care to hospitals); and c) an interprofessional clinicians group. Since its inauguration, the committees have been restructured to be better aligned with priorities and to be more efficient. The committees as of mid-2013 include: a) Standards and Performance Indicators; b) Education and Information; and c) Quality and Best Practices.

## Results

### Early activities and early successes

Since its inception in November 2010, the Board (which meets monthly) and the Regional Program have undertaken numerous activities. These are listed in Table 3. In the first year, the activities focused on the “Year One” Work Plan developed by the Transitional Leadership Group. These activities included activating the Board’s committees and work groups, supporting the Ottawa Hospice Plan to increase residential hospice capacity in Ottawa, establishing standards for the region and performance indicators and implementing a communication and information strategy. Importantly, the Program also became a legally incorporated entity.

**Table 3:** Accomplishments and activities of the Champlain regional HPC program in the first 3 years

Activities	Description
Activities Completed	
Ottawa Hospice Plan Phases 1 and 2	New 10-bed residential hospice in Ottawa and opened hospice in city’s west end.
Service Agreements between the LHIN and HPC Service Providers (entities providing HPC services)	Ensure standards and coordination.
Vetting of Proposals for HPC services to the LHIN	All proposals related to HPC services submitted to the LHIN by various service providers have to be first vetted by the Regional HPC Program Board.
Standards for HPC services across different settings	Standards to guide performance indictor development across sectors created.
Performance Indicators for the Region	22 priority performance indicators have been identified for the region, as well 40 other micro indicators for individual service providers.
Central Referral and Triage System for referrals to Hospices and the Palliative Care Unit (PCU) in Ottawa	One single referral point for patients being referred to Ottawa’s hospices and PCU; can be done online.
Merger of PPSMCS and NPs to establish strong regional community-based consultation and support team (Regional Palliative Consultation Team (RPCT))	(The only region in province to do this).
Expected Death in the Home (EDITH) Protocol	Protocol that allows funeral homes to collect bodies of deceased patients who die at home (expected deaths) without requiring a death certificate to move the body; the death certificate is then completed within 24 h.
Madawaska Rural Program Plan	Rural Hospice Palliative Program in rural and remote south western part of region.
Telelink Project	Video conferencing link ups between all the region’s hospice and palliative care programs for education and telemedicine.
Information Strategy for health professionals and public	Development of a Program website with information for the public, patients and health professionals (http://www.champlainpalliative.ca).
Engaging Family Health Teams Project	Increasing involvement of three out of four family health teams in providing palliative care.
Testing of Emergency Medications and Supplies Box	A rural model and an urban model.
Rural Hospice Palliative Care Retreat	Initiate the process of developing a framework for the implementation of rural- and remote-based HPC.
Framework for Rural and Remote HPC Services	Begin to implement framework.
Coordinated Education Strategy for the region targeting physicians, nurses and pharmacists	Using, amongst others, the Pallium LEAP courseware and online learning modules.
3-Year Work Plan	Work Plan for 2012 to 2015.
Activities Underway	
Palliative and EOL Care in Long-Term Care Framework	
Family Physician Registry	Registry of Family Physicians who provide palliative and end-of-life care for their own patients and those who are interested in taking on new palliative patients.

*EOL Care* End-of-Life Care, *LEAP* Learning Essential Approaches to Palliative and End-of-Life Care, *PPSMCS* Palliative Pain and Symptom Management Consultation Service; now: *RPCT* Regional Palliative Consultation Team, *NP* Nurse Practitioner; in 2012 the Ministry of Health and Long-Term Care allocated 5 NPs to each region, hosted by CCAC

Based on the LHIN-Regional Program MOA, all proposals related to hospice palliative care in the region, including funding requests to the LHIN, are being reviewed by the Regional Program. This provides the Regional Program with the means of coordinating services and implementing a cohesive, priority- and quality-based system in the region. The inaugural Board also worked with the LHIN to modify the service agreements that are signed between the LHIN and HSPs. These service agreements between the LHIN and HSPs articulate the expectations by the LHIN of the HSPs and vice versa, including the expected deliverables. The updated agreements for the HSPs that provide palliative care-related services in the region now require that the HSPs adhere to the standards laid out by the Regional Program, monitor key performance indicators, ensure that their activities are aligned with the Regional Program and the regional plan and have any plans or requests they intend to submit to the LHIN first reviewed and vetted by the Regional Board. This ensures alignment with regional plans and priorities.

### Challenges: past and present

In addition to some early successes, the Regional Program has also experienced expected and unexpected challenges. The main anticipated challenge was the fear by some service providers, including HSPs and some clinicians, that a Regional Program would dilute or remove their locus of control over their own programs and activities. The extensive stakeholder participation in the process leading up to the inauguration of the Regional Program, the terms of reference of the Regional Program (including its governance structure), the MOA and the fact that the Regional Program is not a service provider have helped to ameliorate this perceived threat. While all of the largest service providers were early adopters, a small number of independent health providers have continued to oppose the Program. The Program has continued to engage these health providers and will continue to do so in the future.

A major unanticipated challenge was change in leadership in key stakeholder organizations during the process. These changes were not related to the regionalization process. This required considerable re-engagement and repeated effort by the process leaders to bring the new leads on board and to get their buy-in and support of the process. Communication was an additional challenge. Although anticipated, the scale of communication required had been underestimated, requiring considerable re-investment of effort particularly towards the end of the process. It has also required ongoing communication and attempts at improving dialogue with stakeholders.

There are several ongoing challenges and new threats. Foremost amongst these is a pervasive risk that Board members, when planning or making decisions, may wear “institutional hats” rather than “systems hats”. To mitigate this risk, a declaration of potential conflicts of interests is a first point of order at each Board meeting. Notwithstanding this, the temptation towards wearing “institutional hats” occasionally surfaces. The risk of “micro-management” by the Board of regional activities is ever-present. Instead of focusing on the “larger picture”, some energy has been spent on addressing details, details that are the responsibility of service providers or other entities. One example of this related to the “Clinical Advisory Group”. The role of the group is to identify clinical issues at a regional level and to provide high-level input. At one point the group found itself making specific clinical recommendations on a clinical protocol. Ongoing vigilance for these risks by the Regional Board and Program Office is therefore crucial. The lack of funds to hire a Data Manager to collect, analyze and report on performance metrics was a significant challenge, initially; however, some funds have recently been allocated by the LHIN and the Regional Program to support this critical work in the form of a Decision Support and Data Coordinator.

One of the main threats to the Program is political: the regional structure is based on a close working relationship with the LHIN and with various service providers. The LHIN structures are, however, potentially at risk with any provincial government change. Work is underway to make the Regional Program less vulnerable to such a change and to ensure that a regional, systems-based approach survives government changes in the future.

### Facilitators and enablers

Several enablers have facilitated the implementation of the Regional Program and these merit some attention. The key enablers are listed in Table 4. Previous attempts at establishing regional HPC programs in the city and region, although unsuccessful, had sowed the seeds for this endeavor. The End-of-Life Network Coordinator (GB) was pivotal in providing logistical and project management support. The endorsement by the LHIN Leadership, not only in the form of moral support, but also with start-up funding, was critical. The two co-leads of the process (JP, JC) brought different but complementary competencies and experiences to the process.

**Table 4:** Facilitators and Enablers of Success in Developing a Regional Hospice Palliative Care Program

▪ A history in the region of attempts at initiating a regional program;
▪ A funded full-time coordinator;
▪ Support from the Local Health Integration Network’s (LHIN’s) CEO and Board of Governors;
▪ Starting the process using an Appreciative Inquiry approach;
▪ Early commitment by most stakeholders;
▪ Maintaining and sustaining momentum throughout the process;
▪ Co-chairing of the process by two co-chairs ;
▪ Significant community and stakeholder engagement;
▪ Exemplars in the region of other regional programs, specifically Stroke and Geriatrics-Care of the Elderly;
▪ Use of a “Change Management” approach;
▪ Creation of a common vision early in the process;
▪ Flexibility to adapt and modify the emerging plan and process; and
▪ Establishment of a competency-based Board, instead of one that represents specific service providers, settings and sites of care.

The extensive engagement of stakeholders and the community was time and resource intensive, but appears to have yielded dividends. In addition to engaging the community and benefiting from their insights, it made many individuals and organizations feel included in the plan and facilitated the considerable buy-in achieved. The use of an Appreciative Inquiry approach [13] reduced some of the anxiety of stakeholders; their successes and strengths were recognized, highlighted and valued. Given the significant change that had to occur, managing and facilitating the change was critical. The process leadership regularly re-evaluated the process from the lens of best practices in change management.

Once begun, momentum was sustained throughout the project. This required regular face-to-face and teleconference meetings and frequent communication with stakeholders. The Transitional Leadership Group, for example, met weekly throughout the summer of 2010, even though that period in Canada is usually slower than other times of the year in terms of project activity. Recognizing the complexity of the system, there was flexibility built in to provide an organic process that allowed for modifications and change as further input came in.

## Discussion and reflections

Several models, frameworks and sets of principles exist for managing change and facilitating systems-thinking in the design, redesign and improvement of health care systems. Some are specifically designed for health care systems, while others are adopted and adapted from industry and other fields. It is recognized that although some may represent sequential processes, change management and system redesign requires an iterative, to-and-fro approach. Although the evidence base for these frameworks is variable, they do provide useful reference points with which to reflect on the process undertaken in implementing the Regional Program.

### Systems approach

Systems-thinking as an approach considers the characteristics and effects of complex adaptive systems and “attempts to maximize their positive effects while minimizing unintended negative effects” [14]. Challenges, such as high proportion of cancer patients dying in acute care facilities or excessive use of emergency departments by cancer patients in the last weeks of life, are viewed not as isolated problems, but rather as parts of a wider, dynamic system made up of many components. Deeper understanding of the linkages, relationships, interactions and behaviors among the elements that characterize the entire system are required.

Swanson and colleagues (2012) argue that a comprehensive systems approach should guide planning and development in health practice, education, research and policy [14]. They have identified key ‘systems thinking’ tools and strategies that have the potential for transformational change in health systems. Three overarching themes span these tools and strategies: A) collaboration across disciplines, sectors and organizations; B) ongoing, iterative learning; and C) transformational leadership. Specific strategies include, amongst others: a) developing a shared vision and systems thinking skills among diverse stakeholders through iterative dialogue; b) anchoring collaboration in core values such as equity and patient centeredness; c) taking into consideration the impact of current and new health programmes on existing health systems, avoiding duplication and increasing local ownership and capacity; d) paying attention to social, political and cultural contexts (both current and historical); e) planning for unintended consequences and being willing and ready to adapt; f) engaging stakeholders through regular monitoring and feedback; g) strengthening existing institution and organizations through genuine and equal partnerships; h) developing systems thinking among leaders and managers; and i) embracing self-organizing phenomena that arise naturally.

In a separate paper, Swanson and colleagues (2010) highlighted the importance of taking a holistic view when addressing health system challenges [15]. They argue that a comprehensive systems perspective requires consideration of all individuals and institutions that impact health and their dynamic interactions over time. This holistic approach is relevant for clinical practice, education, research and policy making.

Similarly, Sterman (2006) has highlighted the importance of implementing policies that incorporate simple rules and incentives, allowing local practitioners and others to innovate around health efficiency and quality [16]. The focus should be on high-leverage changes that are likely to have long-term positive effects. One of the approaches the Regional Program is currently adopting is the development of flexible frameworks for the integration of palliative care (including hospices) in rural regions as well as in long-term care facilities in the region. The emerging frameworks provide direction at a high level, particularly with respect to key components and standards (including advance care planning documentation), but leave some room for local adaptations and innovations in the different parts of the region.

The World Health Organization has identified a ten-step approach to systems thinking in health care [17]. These are divided into 2 large phases: I) Intervention Design (Steps 1 to 4) and II) Evaluation Design (Steps 5 to 10). The initial phases include the steps that revolve around convening stakeholders, collective brainstorming, conceptualizing the effects of systems change and, lastly, adapting and redesigning. The second phase follows by determining indicators, choosing methods, selecting designs, developing the plan, setting a budget and identifying the source funding. Upon reflection, we believe the first phase was done relatively well in our Regional Program development process. Clearly we are still in the second phase and are having to periodically revisit some steps in the first phase. The one-year (first year) and three-year work plans identified tasks to be completed and these have guided activities, constituting concrete deliverables. High level systems priority performance indicators have just recently been identified and processes and resources have been put in place to begin to monitor them [18].

Dash, Llewellyn and Richardson (2009) propose that developing a regional health system strategy is an exercise in answering five questions [5]: 1) Why is change necessary?; 2) How will the needs of the population evolve?; 3) What clinical pathways will best meet patients’ future needs?; 4) What delivery models are needed to support optimal care?; and 5) Are the proposed changes affordable and feasible? They also identify four components for achieving change; a) an understanding of and a commitment to change; b) role models to champion the effort; c) formal mechanisms to reinforce change; and d) skills and capabilities for improvement. While the Regional Program process addressed most of these questions and components, it did not explicitly map out the pathways (clinical or access to services). This has recently been identified as a Program priority.

### Change management

Notwithstanding some limitations as highlighted by Appelbaum and colleagues (2012) [19], Kotter’s classical 8-Step change management model provides a good framework to understand change management [20]. The steps include: 1) creating urgency; 2) forming a powerful coalition; 3) creating a vision for change; 4) communicating the vision; 5) removing obstacles; 6) creating short-term wins; 7) building on change; and 8) anchoring the change in corporate culture. In the case of the Regional Program process, there was considerable and deliberate effort put into the first 5 steps. Of particular note is that both of the co-leads (JP, JC) spent a considerable amount of time conducting “shuttle-diplomacy” by meeting with various leaders, managers, agents of change and institutions to make the case, elicit insights and input, which was then used to further crystallize the vision and begin to form the plan. The Regional Program now finds itself in the critical phases of steps 6 to 8. Although there have been some early successes (see Table 4), ongoing attention is required to fully integrate a systems-thinking culture within the Regional Program, the various stakeholders and health care funders and administrators.

Price and Scowcroft (2011) have described essential skills to influence health care system development [21]. These include building relationships, being present, listening, asking the right questions, collecting data, persuading, being flexible and open to change, creating a vision and forming coalitions. Initially, advocacy for the development of a regional systems-based approach fell upon a small number of champions. However, as the process gained momentum, more people and organizations stepped up to this role and a collective advocacy role has evolved. Board members and the Regional Office, as well as many of the stakeholders, are now involved not only in planning and systems evaluation, but also in ongoing engagement and relationship building in the region.

Dickson and colleagues (2012), in a report of the Canadian Health Services Research Foundation, have proposed a non-linear model of key factors for successful change in the health care system in the form of a “Change Map” [22]. In their words: “The change map is an effort to reflect the dynamic, non-linear and interdependent nature of the change process, in a manner consistent with the principles of a complex adaptive system.” The map highlights “territories” to be traversed during change as well as “categories” embedded within the territories. The first “territory” is *Conceptualizing and Preparing*, followed by *Implementation and Sustaining Change*. Within these territories are four categories of change, each with definitions and suggested approaches and tools. The four categories are: 1) Getting ready for change; 2) Implementing change; 3) Spreading change; and 4) Sustaining change. Each of these are defined and broken up into more detailed activities. These activities, for example, include understanding the context and the culture and determining readiness and capacity of change in the first “category”. The main activity in the last category of “sustaining change” highlights the importance of monitoring and assessing change effectiveness and success. As previously mentioned, this latter activity constitutes a current priority for the “young” Regional Program.

## Conclusion

The implementation of the Champlain Hospice Palliative Care Program provides a case study for a systems approach and change management in a health care system that requires collaboration and coordination between many independent service providers. Unlike other jurisdictions in Canada where a single regional health authority is responsible for delivering health services, the system in the province of Ontario has no single service provider. This requires a participative approach to developing regional structures and programs to provide services in specific areas such as hospice palliative care. The Champlain Hospice Palliative Care Program has largely followed best practices as described in various models of change management and health systems implementation. However, it is still a relatively young program and very much in its infancy. The long-term success of the Regional Program depends on its ability to demonstrate measurable benefits. To this end, the custodians of the Program - as well as its funders, stakeholders and partners - strive to maintain the processes set in motion to allow the Program to mature.

## Abbreviations

CCAC, Community Care Access Centre; CHPCP, Champlain Hospice Palliative Care Program; ED, Executive Director; EDITH, Expected Death in the Home; EOL, End-of-Life; HPC, Hospice Palliative Care; HSP, Health Service Provider; LEAP, Learning Essential Approaches to Palliative and End-of-Life Care; LHIN, Local Health Integration Network; LTC, Long-Term Care; MOA, Memorandum of Agreement; MoHLTC, Ministry of Health and Long-Term Care; NP, Nurse Practitioner; PCU, Palliative Care Unit; PPSMCS, Palliative Pain and Symptom Management Consultation Service; RPCT, Regional Palliative Consultation Team
